# A Candidate Gliotransmitter, L-β-Aminoisobutyrate, Contributes to Weight Gain and Metabolic Complication Induced by Atypical Antipsychotics

**DOI:** 10.3390/nu15071621

**Published:** 2023-03-27

**Authors:** Kouji Fukuyama, Eishi Motomura, Motohiro Okada

**Affiliations:** Department of Neuropsychiatry, Division of Neuroscience, Graduate School of Medicine, Mie University, Tsu 514-8507, Japan

**Keywords:** β-aminoisobutyric acid, lurasidone, quetiapine weight gain, AMPK

## Abstract

Lurasidone and quetiapine are effective atypical mood-stabilizing antipsychotics, but lurasidone and quetiapine are listed as lower-risk and high-risk for weight gain/metabolic complications, respectively. The pathophysiology of the discrepancy of metabolic adverse reactions between these antipsychotics remains to be clarified. The GABA isomer, β-aminoisobutyric acid (BAIBA) enantiomer, was recently re-discovered as myokine via an AMP-activated protein kinase activator (AMPK) enhancer and inhibitory gliotransmitter. Notably, activation of AMPK in peripheral organs improves, but in the hypothalamus, it aggravates metabolic disturbances. Therefore, we determined effects of chronic administration of lurasidone and quetiapine on intracellular and extracellular levels of the BAIBA enantiomer. L-BAIBA is a major BAIBA enantiomer in the hypothalamus and astrocytes, whereas L-BAIBA only accounted for about 5% of total plasma BAIBA enantiomers. Chronic lurasidone administration did not affect body weight but decreased the L-BAIBA level in hypothalamus and cultured astrocytes, whereas chronic quetiapine administration increased body weight and the L-BAIBA level in hypothalamus and astrocytes. Contrary, neither lurasidone nor quetiapine affected total plasma levels of the BAIBA enantiomer since D-BAIBA levels were not affected by these antipsychotics. These results suggest that activation of intracellular L-BAIBA signaling is, at least partially, involved in the pathophysiology of metabolic adverse reaction of quetiapine. Furthermore, this study also demonstrated that lurasidone and quetiapine suppressed and enhanced astroglial L-BAIBA release induced by ripple-burst stimulation (which physiologically contributes to cognitive memory integration during sleep), respectively. Therefore, L-BAIBA probably contributes to the pathophysiology of not only metabolic adverse reactions, but also a part of clinical action of lurasidone or quetiapine.

## 1. Introduction

Second generation antipsychotics (atypical antipsychotics) have provided the improvement of quality of life of individuals with schizophrenia via controlling positive and mainly negative symptoms; however, currently, psychiatry has faced another unexpected issue, such as higher prevalence of metabolic disturbance in schizophrenia. Indeed, about 40% of patients with schizophrenia are suffering with metabolic disturbance [1,2]. It has been considered that atypical antipsychotics play important roles in the pathophysiology of metabolic complications in patients with schizophrenia, since after the approval of atypical antipsychotics, the mortality gaps between individuals with schizophrenia and the general population have been amplifying [3,4]. The life expectancy of individuals with schizophrenia is more than 15 years shorter than the general population, and it is speculated that metabolic complications possibly contribute to approximately 30% for excess deaths [3,4,5].

Recent hypothetical pathophysiology of antipsychotics-induced metabolic complication has considered that the inhibition of histamine H1 and serotonin 5-HT2A receptors indirectly disturbs the energy regulation systems in the hypothalamus [3,6]. Detailly, activation of the H1 and 5-HT2A receptors enhance inositol trisphosphate (IP3) synthesis, which is an endogenous inducer for the calcium-induced calcium releasing system (CICR) via stimulation of the IP3 receptor [7,8,9]. Decreased CICR secondarily suppresses synthesis of adenosine triphosphate (ATP), leading to activation of signaling of adenosine-monophosphate (AMP)-activated protein kinase (AMPK) [3,6,10,11]. This 5-HT2A/H1 hypothesis is supported by the clinical findings that high affinity H1 and 5-HT2A receptor antagonistic antipsychotics, including zotepine, quetiapine, olanzapine, and clozapine are listed in the high-risk for weight gain and metabolic complication [4].

It has been established that activation of AMPK is one of the major targets for treating insulin-resistant diabetes [12,13]. In fact, a meta-analysis study reported that clozapine-induced weight gain was meaningfully suppressed by metformin (AMPK activator) [14]. AMPK regulates metabolism in peripheral organs, whereas AMPK in the hypothalamus is a fundamental player for regulation of both sides in the energy balance equation (feeding and energy expenditure) in the body [12]. Other recent meta-analyses reported that antipsychotics-induced dose-response weight gain is composed of three patterns: monotonic (dose-dependently increased weight gain, such as clozapine), biphasic (dose-dependently increased within therapeutic-relevant dose range, but a supratherapeutic dose conversely decreased weight, such as quetiapine), and hyperbolic (increased weight dose-dependently but existence of a plateau phase, such as the majority of antipsychotics) [4,15]. These clinical implications suggest that the pathophysiology of atypical antipsychotic-induced weight gain is probably attributed to the interaction of contradictory mechanisms of weight-gain-enhancing and suppressing systems.

Our recent study reported the possibilities that the pathophysiology of clozapine-induced weight gain involves direct regulation of AMPK via L-β-aminoisobutyric acid (L-BAIBA) and 5-HT7 receptor, in addition to an indirect activation pathway through 5-HT2A/H1 receptor inhibition [11]. The BAIBA enantiomer (GABA isomer) recently has been re-discovered as protective myokine, regulating adipose tissue browning, it enhances sensitivity to insulin, and improves obesity induced by a high-fat diet [16,17,18]. Additionally, the BAIBA enantiomer increases the signaling of insulin receptor substrate, AMPK, Akt, and decreases the expression of gluconeogenic enzymes [18]. Clozapine, the high-risk antipsychotics for weight gain and metabolic complication [4,15], enhanced AMPK signalings via increasing BAIBA synthesis, whereas brexpiprazole, the lower-risk antipsychotics for weight gain [4,15], conversely supressed AMPK signalings via decreasing BAIBA synthesis [11]. These discrepancies between brexpiprazole and clozapine can provide a candidate hypothetical pathophysiology of antipsychotics-induced weight gain that increasing BAIBA plays important roles in the antipsychotics-induced activation of AMPK signalings. On the contrary, inhibition of 5-HT7 receptor suppresses AMPK signalings via decreasing cAMP synthesis [6,19,20,21]. Considering these preclinical findings, the interaction between stimulatory effects of increasing L-BAIBA and inhibitory effects of 5-HT7 receptor inhibition on AMPK signalings contributes to the bell-shaped dose–response curve of weight gain induced by several atypical antipsychotics, such as quetiapine [4,15]. On the contrary, if lurasidone, with the lowest-risk for weight gain and metabolic complications [4,15], decreases or does not affect intracellular L-BAIBA levels, similar to brexpiprazole [11], the pathophysiology of low-risk for weight gain of lurasidone can also be explained. To clarify our hypothesis, the present study determined the effects of chronic administrations of therapeutic-relevant and supratherapeutic dose/concentration of lurasidone and quetiapine on the intracellular levels of the BAIBA enantiomer.

Furthermore, in the central nervous system, L-BAIBA is a dominant enantiomer and activates glycine, GABA_A_, and GABA_B_ receptors [11,22,23]. Therefore, L-BAIBA probably contributes to the pathophysiology of not only adverse reactions, such as weight gain and metabolic complication, but also clinical mood stabilizing and/or antipsychotic effects via these transmitter receptors modulation. Based on these preclinical findings, the present study also determined the extracellular levels of L-BAIBA in the hypothalamus using microdialysis and astroglial L-BAIBA release, using cultured astrocytes to explore the release mechanisms of L-BAIBA.

## 2. Materials and Methods

### 2.1. Experimental Animals

Any procedures in this report including animal care were conducted according to the ethical guidelines established by the Institutional Animal Care and Use Committee at Mie University, Japan (No.2019-3, 24 May 2019) and are reported in accordance with the ARRIVE (Animal Research: Reporting of In Vivo Experiments) guidelines [24]. Male Sprague-Dawley rats (SLC, Shizuoka, Japan: ranged from 6 to 8 weeks of age: *n* = 66) were used for in vivo (body weight, microdialysis and intracellular levels of transmitter and messenger in the hypothalamus). Neonatal Sprague-Dawley rats (within 48 h after birth: *n* = 24, SLC) were used for in vitro primary cultured cortical astrocytes studies. Rats were individually housed in cages with air-conditioning rooms (22 ± 2 °C) set at 12 h light and dark cycle, and given free access to food or water.

### 2.2. Chemical Agents

Lurasidone was obtained from FUJIFILM-Wako (Osaka, Japan). The 5-HT7 receptor inverse-agonist, SB269970, was obtained from Cosmo-Bio (Tokyo, Japan). Quetiapine, vigabatrin, inhibitor of 4-aminobutyrate aminotransferase (ABAT), which is well known for being responsible for the catabolism of γ-aminobutyric acid (GABA), carbenoxolone (hemichannel and gap-junction suppressor), and L-β-aminoisobutyric acid (L-BAIBA) were obtained from Funakoshi (Tokyo, Japan). All compounds used in this study were prepared on the day of the experiment. Lurasidone and quetiapine were dissolved in dimethyl sulfoxide (DSMO) with 1 mM cinnamic acid [20]. The final concentration of DMSO was lower than 0.1% (*v*/*v*). Vigabatrin, SB269970, and carbenoxolone were directly dissolved in an experimental medium.

Therapeutic-relevant and supratherapeutic serum concentrations of lurasidone are ranged from 30–100 nM and higher than 500 nM, respectively [25,26]. Therapeutic-relevant and supratherapeutic serum concentrations of quetiapine ranged from 0.3–10 μM and higher than 30 μM, respectively [19,25,26,27]. Based on these clinical findings and preclinical demonstrations, in this study, primary cultured cortical astrocytes were chronically (for 14 d) exposed to supratherapeutic concentration of lurasidone (500 nM) and quetiapine (30 μM) and therapeutic-relevant concentration of lurasidone (100 nM) and quetiapine (3 μM) [19,20,27,28]. Previously, pharmacodynamic studies reported that the effective dose of systemic administration of lurasidone [29,30] and quetiapine [31] to schizophrenic models were 1 and 10 mg/kg, respectively. Based on these previous studies, rats were chronically administered lurasidone (1 and 3 mg/kg/day) and quetiapine (10 and 30 mg/kg/day) for 14 d, using subcutaneously osmotic pumps (2ML_1; Alzet, Cupertino, CA, USA) [19,20].

To suppress ABAT, astrocyte was exposed to 200 μM vigabatrin for 14 d [32] and rats were subcutaneously administered by vigabatrin (75 mg/kg/day for 14 d) [33]. To inhibit astroglial hemichannel, in cultured astrocyte and microdialysis studies, 100 μM carbenoxolone was acutely applied [11,34].

### 2.3. Primary Cultured Astrocytes

The procedures of primary cultured cortical astrocytes were performed in accordance with our previous reports [35,36]. Cortical astrocyte was prepared from neonatal rats (0~48 h of age). Under the dissecting microscope, the cerebral hemisphere was removed. Suspension of brain tissues were filtered through 70 µm nylon-mesh (BD, Franklin Lakes, NJ, USA). The pellet of cortical tissue was suspended in Dulbecco’s modified Eagle’s medium containing 10% of foetal calf serum (fDMEM). After 14 d of culture (DIV14) to DIV28, astrocytes were trypsinized and seeded directly on a translucent polyethylene terephthalate membrane (1.0 μm) with 24 well plates (BD, Franklin Lakes, NJ) at a density of 100 cells/cm^2^ for experiments. To study the effects of chronic administration of therapeutic-relevant and supratherapeutic concentrations of lurasidone and quetiapine on BAIBA enantiomers, GABA and D-serine, astrocytes were incubated in fDMEM containing lurasidone (100 and 500 nM) [27,37], quetiapine (3 and 30 μM) [19], vigabatrin (200 μM), vigabatrin with lurasidone or quetiapine [11,32] for 14 d (DIV14-28). fDMEM was changed twice a week.

On DIV28, astrocyte was incubated in artificial cerebrospinal fluid (ACSF) (150 mM Na^+^, 3.0 mM K^+^, 1.4 mM Ca^2+^ and 0.8 mM Mg^2+^ and 5.5 mM glucose adjusted to pH = 7.3 using 20 mM HEPES buffer) containing the same target drugs, without or with 100 μM carbenoxolone, at 35 °C for 120 min. During resting stages, hemichannel mainly does not function due to their low open probabilities [38]. Therefore, to study the functions of activated hemichannels in physiological conditions, the cultured cortical astrocytes were stimulated by ripple-burst stimulation using a busdrive amplifier (SEG-3104MG; Miyuki Giken, Tokyo, Japan). Ripple-burst-evoked stimulation is generally synchronized with sleep spindles during the non-REM sleep phase by wide-band electrocorticogram [38]. It has been well known that ripple-burst plays an important role in sleep-dependent memory integration as a cognitive component [39]. Ripple-burst-evoked stimulation was set at square-wave direct-current pulse output with a magnitude of 300 mV/mm^2^ [38]. A ripple-burst-evoked stimulation was composed of 10 stimuli at 200 Hz and 10 bursts (50% duty cycle) at burst intervals of 100 ms/s [38]. The pattern of ripple-bursts was generated using LabChart version 8.2 software (AD Instruments, Dunedin, New Zealand).

### 2.4. Microdialysis

Rats were anaesthetised with 1.8% isoflurane on stereotaxic frame for 1 h [40]. A concentric direct insertion-type microdialysis probe (1 mm exposed membrane: Eicom, Kyoto, Japan) was inserted in the hypothalamus, mainly ventromedial nucleus of the hypothalamus (A = −3.2 mm, L = +0.5 mm, V = −9.2~−10.2 mm, relative to bregma; 0.22 mm diameter) [11]. AMPK in the ventromedial hypothalamic nucleus regulates glucose and lipid metabolism via modulating thermogenesis in brown adipose tissue, function and browning in white adipose tissue [12]. After the surgery, rats were housed in cages individually for recovery. During the experiments, rats were provided food and water ad libitum.

After 18 h recovery from the anaesthesia, the MRS perfusion was started. Rats were placed into freely moving animal system individually (Eicom) equipped with swivel system (TCS2-23; ALS, Tokyo, Japan). Perfusion rate was maintained at 2 μL/min using modified Ringer solution (MRS) (145 mM Na^+^, 2.7 mM K^+^, 1.2 mM Ca^2+^, 1.0 mM Mg^2+^, and 154.4 mM Cl^−^ adjusted to pH = 7.4 using 2 mM phosphate buffer and 1.1 mM Tris buffer) or 100 mM K^+^ containing MRS (HKMRS) (47.7 mM Na^+^, 100 mM K^+^, 1.2 mM Ca^2+^ and 1.0 mM Mg^2+^) [41]. Perfusate was collected every 20 min. Transmitter levels were monitored, 8 h after starting perfusion with MRS. To analyze the releases of GABA and BAIBA enantiomers induced by depolarisations in the hypothalamus, the perfusate was switched from MRS with or without (control) 100 μM carbenoxolone [37] to HKMRS containing the same agent for 20 min (HKMRS-evoked stimulation).

Collected perfusates were injected to ultra-high-performance liquid chromatography (UHPLC) by an autosampler (xLC3059AS; Jasco, Tokyo, Japan). After the microdialysis study, inserting locations of the microdialysis probe was verified by preparing 200 μm thick brain tissue slices (Vibratome 1000; Technical Products, St. Louis, MO, USA).

### 2.5. Capillary Immunoblotting Analysis

Protein expression level was determined using capillary immunoblotting system (Wes, ProteinSimple, Santa Clara, CA, USA). The procedures were mainly in accordance with the manufacturer instructions (ProteinSimple). Extracted lysates of hypothalamus and cultured astrocyte were mixed with the master mix (ProteinSimple), and then heated at 95 °C for 5 min. All samples and reagents, such as primary antibodies, horseradish peroxidase (HRP)-conjugated secondary antibodies, blocking reagents, chemiluminescent substrate (SuperSignal West Femto; Thermo Fisher Scientific, Waltham, MA, USA), and separation and stacking matrices were dispensed into a designated 25 well plate [42]. Electrophoresis/immunodetection were performed by an automated capillary system. Briefly, capillaries were incubated in a blocking reagent for 15 min, and target proteins were probed with primary antibodies, followed by incubation with HRP-conjugated secondary antibodies (Anti-Rabbit IgG HRP, A00098, 10 μg/mL; GenScript, Piscataway, NJ, USA). Antibodies against AMPKα (2603, 1:50; Cell Signalling, Danvers, MA, USA), phosphorylated AMPKα (2535, 1:50; Cell Signalling) and GAPDH (NB300-322, 1:100, Novus Biologicals, Littleton, CO, USA) were diluted in an antibody diluent (ImmunoShot Platinum, Cosmo Bio, Tokyo, Japan) [42].

### 2.6. Extractions

Hypothalamic tissues were dissected [43], after chronic administration of target drugs (lurasidone, quetiapine or vigabatrin) for 14 days. To determine the AMPK signaling using Wes (capillary immunoblotting), hypothalamic tissue and astrocytes were extracted using Minute Plasma Membrane Protein Isolation Kit (Invent Biotechnologies, Plymouth, MN, USA). Total protein levels were analyzed using Protein Assay Reagent kit (FUJIFILM-Wako Pure Chemical Corporation, Osaka, Japan).

To measure the concentrations of the BAIBA enantiomer, GABA, D-serine, cAMP, AMP, IP3, and ATP in the hypothalamus and astrocytes, hypothalamus, and astrocyte were homogenized with an ultrasonic cell disrupter (VP-050N, Taitec, Koshigaya, Japan) in chilled 4 N perchloric acid with 4.3 mM EDTA [44]. The extracted samples were centrifuged at 10,000× *g* for 20 min at 4 °C, and then, 5 μL filtered aliquots were injected into UHPLC or UHPLC equipped with mass spectrometry (UHPLC-MS).

### 2.7. UHPLC and UHPLC-MS

Dually derivatized with amino-acidic transmitters, including BAIBA enantiomers, serine enantiomer and GABA with o-phthalaldehyde and isobutyryl-L-cysteine were separated by UHPLC (xLC3185PU, Jasco, Tokyo, Japan) [45]. Briefly, derivatives were prepared by a 5 μL sample and dual-derivative reagent in reaction wells for 5 min. The derivatized sample (5 μL) was injected by an autosampler (xLC3059AS; Jasco). The flow rate of mobile phases was set at 0.5 mL/min, and the analytical column (Triat C18, particle size: 1.8 µm, 50 mm × 2.1 mm; YMC, Kyoto, Japan) was set at 35 °C. The gradient elution program was performed over 15 min with mobile phases A (0.1 M citric acid buffer, pH = 3.5) and B (acetonitrile). Wavelengths for excitation/emission in the fluorescence detector (xLC3120FP, Jasco) were set to 345/455 nm, respectively.

Levels of IP3, ATP, cAMP, and AMP were measured using UHPLC-MS (Acquity UHPLC H-Class equipped with Acquity SQ detector; Waters, Milford, MA, USA). The extracted sample (5 μL) was injected using an autosampler (Acquity UHPLC Sample Manager FTN; Waters), and separated by a graphite carbon column (particle size: 3 μm, 150 × 2.1 mm; Hypercarb, Thermo) maintained at 0.4 mL/min at 40 °C. To monitor the levels of AMP, cAMP, and ATP, the linear gradient elution program was used for over 10 min with mobile phases A (1 mM ammonium/acetate buffer, pH = 11) and B (acetonitrile) [11]. Nitrogen flow rates of the cone and desolvation were set at 5 and 750 L/h, respectively. The temperature for desolvation was set to 450 °C. Cone voltages for the measurement of AMP (*m*/*z* = 348.2), ATP (*m*/*z* = 508.2) and cAMP (*m*/*z* = 330.3), were 40, 34, and 42 V, respectively.

To monitor the IP3 level, the linear gradient elution program was used for over 10 min with mobile phases A (10% acetate) and B (100% acetonitrile). The flow rates of nitrogen for desolvation/cone were set at 750/5 L/h, respectively. The temperature for desolvation was set to 450 °C. The cone voltage used to determine IP3 (*m*/*z* = 421.1) was 35 V.

### 2.8. Data Analysis

Any experiments were designed with equal group sizes (number: *n* = 6) without conducting power analyses, according to previous reports. Especially, regarding in vitro experiments using cultured astrocytes, one group contains data from six independent rats, but not composed the data from astrocytes extracted from the same individual. Any data are expressed mean ± standard-deviation (SD), and two-tailed *p* < 0.05 was considered statistically significant. Drug level for chronic administration was selected based on previous studies [21,27,33,35,46]. To measure levels of GABA, BAIBA enantiomer, D-serine, IP3, AMP, cAMP, ATP, and protein expression, the sample orders on Wes and autosamplers were selected using random numbers.

Effects of chronically administration of lurasidone, quetiapine and vigabatrin on concentrations of GABA, BAIBA enantiomers, D-serine, IP3, cAMP, AMP, ATP, and AMPK were analyzed by analysis of variance (ANOVA) with Tukey’s post hoc test or student T-test using BellCurve for Excel version 3.2 (Social Survey Research Information Co., Ltd., Tokyo, Japan). Effects of chronically administration of lurasidone, quetiapine, and vigabatrin on extracellular levels of D-serine and BAIBA enantiomers were assessed using multivariate ANOVA (MANOVA) with Tukey’s post hoc test (BellCurve for Excel). When the data did not violate the assumption of sphericity (*p* > 0.05), F-value of MANOVA was analyzed using sphericity-assumed degrees of freedom. However, if the assumption of sphericity was violated (*p* < 0.05), the F-value was analyzed using chi-Muller’s corrected degrees of freedom. When F-value for any factors in MANOVA was significant, the data were analysed using Tukey’s post hoc test. The rat body weight between before and after chronic administration of vehicle (control), lurasidone (1 and 3 mg/kg/day for 14 d), and quetiapine (3 and 30 mg/kg/day for 14 d) were analyzed by ANOVA with Tukey’s post hoc test.

## 3. Results

### 3.1. Body Weight

**Figure 1:**
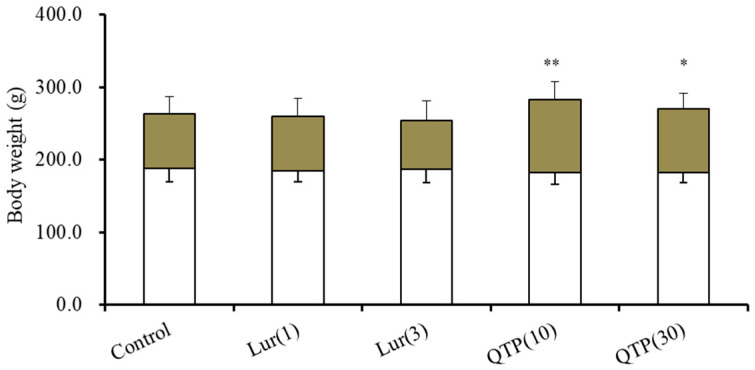
Impacts of chronic administration of quetiapine (10 and 30 mg/kg/day for 14 d) and lurasidone (1 and 3 mg/kg/day for 14 d) on rat body weight. Ordinate indicates mean ± standard deviation (SD) (*n* = 6) of rat body weight (g). Opened and brown columns indicate the rat body weight before (6 weeks of age) and after (8 weeks of age) the chronic administration of antipsychotics, respectively. *: *p* < 0.05, **: *p* < 0.01: relative to control (vehicle alone: MRS containing 0.1% DMSO) by analysis of variance (ANOVA) with Tukey’s post hoc test.

Chronic administration of lurasidone (1 or 3 mg/kg/day for 14 d) did not affect the body weight, whereas quetiapine (10 and 30 mg/kg/day for 14 d) increased the body weight compared with control rats (F(2,15) = 13.1(*p* < 0.01)) (Figure 1).

### 3.2. In Vivo Experiments

#### 3.2.1. Plasma BAIBA Enantiomer Levels

**Figure 2:**
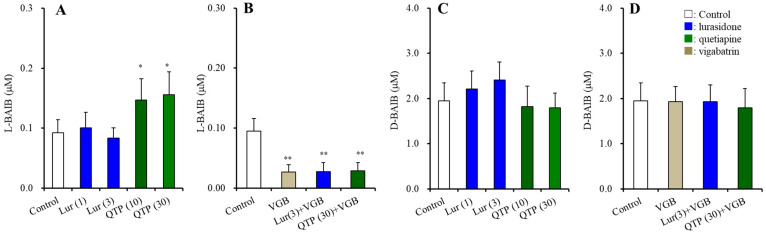
Effects of chronic administration of lurasidone (Lur: 1 and 3 mg/kg/day for 14 d), quetiapine (QTP: 10 and 30 mg/kg/day for 14 d), and chronic co-administration of vigabatrin (VGB: 75 mg/kg/day) with lurasidone (3 mg/kg/day) or quetiapine (30 mg/kg/day) for 14 d on plasma levels of BAIBA enantiomers, L-BAIBA (**A**,**B**) and D-BAIBA (**C**,**D**). Ordinate: mean ± SD (*n* = 6) of plasma levels of BAIBA enantiomers (μM). *: *p* < 0.05, **: *p* < 0.01: relative to control (vehicle alone: MRS containing 0.1% DMSO) by ANOVA with Tukey’s post hoc test.

Plasma concentrations of L-BAIBA and D-BAIBA in antipsychotics naive rats (control) were 0.09 ± 0.02 μM and 1.95 ± 0.40 μM, respectively (Figure 2). These results suggest that D-BAIBA is a dominant BAIBA enantiomer (>95%) in the blood.

The plasma level of D-BAIBA was not affected by chronic administration of lurasidone (1 and 3 mg/kg/day for 14 d) or quetiapine (10 and 30 mg/kg/day for 14 d) (Figure 2C). The plasma L-BAIBA level was not affect by chronic administration of lurasidone, but was increased by quetiapine (F(2,15) = 6.6 (*p* < 0.01)) (Figure 2A). The effect of the ABAT inhibitor, vigabatrin (75 mg/kg/day for 14 d) on plasma levels of the BAIBA enantiomer was determined, since ABAT is a rate-limiting synthesised enzyme of L-BAIBA [11,47,48]. Chronic administration of vigabatrin drastically decreased the plasma L-BAIBA level (*p* < 0.01), but did not affect the D-BAIBA level (Figure 2B,D). After the chronic co-administration of vigabatrin with quetiapine for 14 d, the stimulatory effects of quetiapine on plasma L-BAIBA level were not observed (Figure 2B).

#### 3.2.2. BAIBA Enantiomer Levels in the Hypothalamus

**Figure 3:**
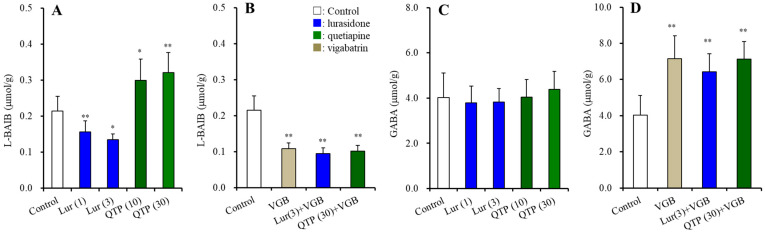
Effects of chronic administration of lurasidone (Lur: 1 and 3 mg/kg/day for 14 d), quetiapine (QTP: 10 and 30 mg/kg/day for 14 d), and co-administration of vigabatrin (VGB: 75 mg/kg/day) with lurasidone (3 mg/kg/day) or quetiapine (30 mg/kg/day) for 14 d on hypothalamic levels of L-BAIBA (**A**,**B**) and GABA (**C**,**D**). Ordinates indicate mean ± SD (*n* = 6) of levels of L-BAIBA or GABA in the rat hypothalamus (μmol/g protein). *: *p* < 0.05, **: *p* < 0.01: relative to control by ANOVA with Tukey’s post hoc test.

Hypothalamic L-BAIBA concentrations were 0.22 ± 0.04 μmol/g protein, whereas D-BAIBA level could not be detected (Figure 3) [47,48]. Chronic administration of vigabatrin (ABAT inhibitor) decreased and increased the levels of L-BAIBA and GABA, respectively (*p* < 0.01) (Figure 3B,D). Chronic lurasidone administration decreased L-BAIBA levels (F(2,15) = 11.0 (*p* < 0.01)) but did not affect levels of GABA (Figure 3A,C). Contrary to lurasidone, chronic quetiapine administration increased the L-BAIBA level (F(2,15) = 6.7 (*p* < 0.01)) but did not affect the GABA level (Figure 3A,C). Chronic co-administration of vigabatrin with lurasidone or quetiapine abolished the decreasing by lurasidone and increasing by quetiapine of the L-BAIBA level (Figure 3B,D).

These results suggest that L-BAIBA is possibly the major BAIBA enantiomer in the hypothalamus. Neither the inhibitory effect of lurasidone nor the stimulatory effects of quetiapine on L-BAIBA level were modulated by ABAT activity.

#### 3.2.3. Effects of Chronic Administration of Lurasidone and Quetiapine on Intracellular Levels of Second Messengers in the Hypothalamus

**Figure 4:**
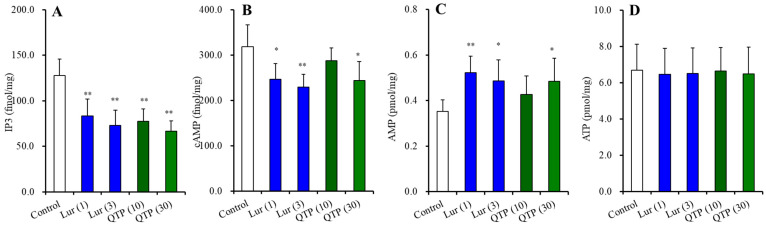
Effects of chronic administration of lurasidone (Lur: 1 and 3 mg/kg/day for 14 d), quetiapine (QTP: 10 and 30 mg/kg/day for 14 d) on intracellular levels of IP3 (**A**), cAMP (**B**), AMP (**C**), and ATP (**D**) in the hypothalamus. Ordinates indicate mean ± SD (*n* = 6) of levels of IP3, cAMP (fmol/mg) and AMP, ATP (pmol/mg) in the rat hypothalamus. *: *p* < 0.05, **: *p* < 0.01: relative to control by ANOVA with Tukey’s post hoc test.

Chronic administrations of lurasidone (F(2,15) = 16.1 (*p* < 0.01)) and quetiapine (F(2,15) = 29.6 (*p* < 0.01)) decreased the intracellular IP3 level in the hypothalamus (Figure 4A). Chronic administrations of lurasidone also decreased the intracellular cAMP level (F(2,15)= 9.2 (*p* < 0.01)), whereas 30 mg/kg/day quetiapine decreased but 10 mg/kg/day quetiapine did not affect the cAMP level in the hypothalamus (F(2,15) = 5.1 (*p* < 0.05)) (Figure 4B). Chronic administrations of lurasidone increased intracellular AMP level (F(2,15) = 9.1 (*p* < 0.01)), whereas 30 mg/kg/day quetiapine also increased but 10 mg/kg/day quetiapine did not affect the AMP level in the hypothalamus (F(2,15) = 4.1 (*p* < 0.05)) (Figure 4C). Neither chronic administrations of lurasidone nor quetiapine affected the intracellular ATP level (Figure 4D).

#### 3.2.4. L-BAIBA Release in the Hypothalamus

Extracellular BAIBA functions in the central nervous system have been already discovered as agonists of glycine, GABA_A_, and GABA_B_ receptors [11,22,23]. We have already demonstrated that L-BAIBA was mainly released from astrocytes [11,49]. Therefore, to clarify whether the inhibitory and stimulatory effects of respective lurasidone and quetiapine on intracellular levels of L-BAIBA in the hypothalamus are reflected in L-BAIBA release, the effects of chronic administration of effective doses of lurasidone (1 mg/kg/day for 14 d) and quetiapine (10 mg/kg/day for 14 d) on HKMRS-evoked L-BAIBA releases were determined using in vivo microdialysis.

**Figure 5:**
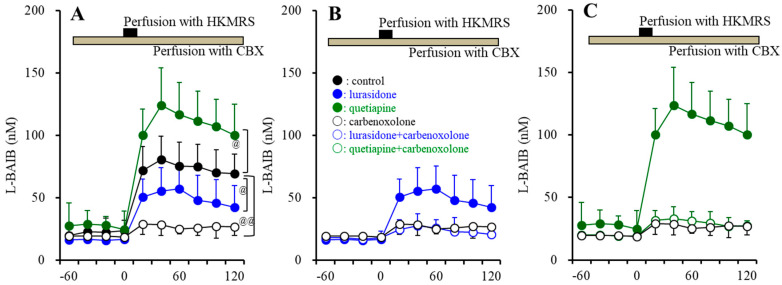
Effects of chronic administration of effective doses of lurasidone ((**B**): Lur: 1 mg/kg/day for 14 d), quetiapine ((**C**): QTP: 10 mg/kg/day for 14 d), and perfusion with 100 μM carbenoxolone (CBX: hemichannel inhibitor) on HKMRS-evoked L-BAIBA release in the hypothalamus using microdialysis. Ordinates indicate mean ± SD (*n* = 6) of extracellular L-BAIBA level (nM). Abscissas indicate time after HKMRS-evoked stimulation (100 mM K^+^ containing perfusate) (min). Black and grey columns indicate the perfusion with HKMRS for 20 min and perfusion with carbenoxolone (100 μM) containing perfusate into the hypothalamus, respectively. @: *p* < 0.05, @@: *p* < 0.01: relative to control (**A**) by multivariate analysis of variance (MANOVA) with Tukey’s post hoc test.

Basal extracellular L-BAIBA level (19.9 ± 8.7 nM) was detected, but that of D-BAIBA could not be detected in the hypothalamus by microdialysis (Figure 5). Usually, to monitor the depolarization-induced transmitter release using microdialysis, perfusion with high K^+^-containing medium (ranged from 25–50 mM K^+^) has been adopted [7,50,51]. However, these conventional K^+^-evoked stimulations (25–50 mM) could not increase the extracellular L-BAIBA level [9]. Contrary, the HKMRS-evoked stimulation (100 mM K^+^) drastically increased the extracellular L-BAIBA level, whereas the extracellular level of D-BAIBA remained not detectable. Perfusion with 100 μM carbenoxolone (hemichannel inhibitor) prevented HKMRS-evoked releases of L-BAIBA (F_time_(5,50) = 3.54 (*p* < 0.01), F_carbenoxolone_(1,10) = 36.9 (*p* < 0.01), F_time*carbenoxolone_(5,50) = 2.9 (*p* < 0.05)) (Figure 5A). Chronic administration of lurasidone (1 mg/kg/day for 14 d) (F_time_(5,50)= 10.7 (*p* < 0.01), F_lurasidone_(1,10)= 5.7(*p* < 0.01), F_time*lurasidone_(5,50) = 1.4 (*p* > 0.05)) and quetiapine (10 mg/kg/day for 14 d) (F_time_(5,50) = 10.4 (*p* < 0.01), F_quetiapine_(1,10) = 9.3 (*p* < 0.01), F_time*quetiapine_(5,50) = 2.1 (*p* > 0.05)) decreased and increased HKMRS-evoked releases of L-BAIBA, respectively (Figure 5A). During the perfusion with 100 μM carbenoxolone, the stimulatory effect of quetiapine and the inhibitory effect of lurasidone on HKMRS-evoked L-BAIBA release were not observed (Figure 5B,C).

#### 3.2.5. Intracellular and Extracellular Levels of D-serine in the Hypothalamus

The effects of lurasidone and quetiapine on L-BAIBA release were speculated to be mediated by both metabolism and hemichannel activity since these antipsychotics affected the intracellular levels of L-BAIBA and hemichannel turnover [6,19,20,27]. Therefore, to clarify whether the inhibitory and stimulatory effects of respective lurasidone and quetiapine on HKMRS-evoked L-BAIBA release in the hypothalamus are reflected in astroglial non-exocytosis release through an activated hemichannel, the effects of chronic administration of lurasidone (1 mg/kg/day for 14 d) and quetiapine (10 mg/kg/day for 14 d) on HKMRS-evoked release of D-serine, which is an established astroglial transmitter [19,35,36,37], were examined.

**Figure 6:**
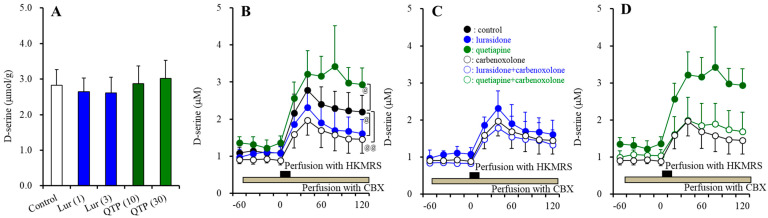
Effects of systemic chronic administration of effective doses of lurasidone (Lur: 1 mg/kg/day for 14 d) and quetiapine (QTP: 10 mg/kg/day for 14 d) on intracellular (**A**) and extracellular (**B**–**D**) levels of D-serine. In panel (**A**), ordinate indicates mean ± SD (*n* = 6) of intracellular levels of D-serine (μmol/g protein). In panels (**B**–**D**), ordinate indicates mean ± SD (*n* = 6) of extracellular levels of D-serine (μM). Abscissa indicates time after HKMRS-evoked stimulation (100 mM K^+^ containing perfusion medium) (min). Black and grey columns indicate the perfusion with HKMRS for 20 min and perfusion with 100 μM carbenoxolone (CBX) containing perfusate, respectively. @: *p* < 0.05, @@: *p* < 0.01: relative to control by MANOVA with Tukey’s post hoc test.

In the hypothalamus, the intracellular D-serine level was 2.82 ± 0.44 μmol/g protein, (Figure 6A). Neither chronic administrations of lurasidone (1 mg/kg/day for 14 d) nor quetiapine (10 mg/kg/day for 14 d) affected the intracellular D-serine release in the hypothalamus (Figure 6A). HKMRS-evoked stimulation drastically increased the extracellular D-serine level (Figure 6B). Perfusion with 100 μM carbenoxolone (hemichannel inhibitor) inhibited HKMRS-evoked D-serine release (F_time_(5,50) = 32.6 (*p* < 0.01), F_carbenoxolone_(1,10) = 8.8 (*p* < 0.01), F_time*carbenoxolone_(5,50) = 1.2 (*p* > 0.05)) (Figure 6B). Chronic administration of lurasidone (1 mg/kg/day for 14 d) (F_time_(5,50) = 36.8 (*p* < 0.01), F_lurasidone_(1,10) = 5.2 (*p* < 0.05), F_time*lurasidone_(5,50) = 1.9 (*p* > 0.05)) and quetiapine (10 mg/kg/day for 14 d) (F_time_(1.6,16.3) = 9.8 (*p* < 0.01), F_quetiapine_(1,10) = 5.8 (*p* < 0.05), F_time*quetiapine_(1.6,16.3) = 3.3 (*p* < 0.05)) decreased and increased HKMRS-evoked releases of D-serine, respectively (Figure 6B). During the perfusion with 100 μM carbenoxolone, the stimulatory effect of quetiapine and the inhibitory effect of lurasidone on HKMRS-evoked D-serine release could not be observed (Figure 6C,D).

### 3.3. In Vitro Experiments

#### 3.3.1. BAIBA Enantiomer Levels in the Astrocyte

**Figure 7:**
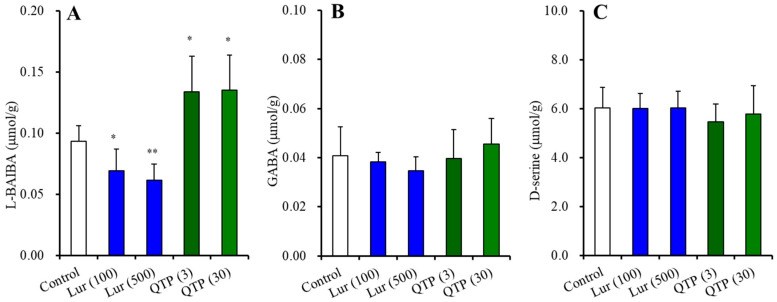
Effects of chronically exposure of lurasidone (Lur: 100 and 500 nM for 14 d), quetiapine (QTP: 3 and 30 μM for 14 d) on intracellular levels of L-BAIBA (**A**), GABA (**B**), and D-serine (**C**) in the astrocytes. Ordinate: mean ± SD (*n* = 6) of levels of L-BAIBA, GABA and D-serine in the astrocytes (μmol/g protein). *: *p* < 0.05, **: *p* < 0.01: relative to control by ANOVA with Tukey’s post hoc test.

In the primary cultured cortical astrocytes, the levels of L-BAIBA, GABA and D-serine were 0.093 ± 0.013, 0.041 ± 0.012 and 6.04 ± 0.83 μmol/g protein, respectively (Figure 7); however, the D-BAIBA level could not be detected.

Chronic exposures of lurasidone (100 and 500 nM) (F(2,15) = 7.7 (*p* < 0.01)) and quetiapine (3 and 30 μM) [F(2,15) = 5.6 (*p* < 0.05)] decreased and increased intracellular L-BAIBA levels in the astrocytes, respectively (Figure 7A). In contrast, neither lurasidone nor quetiapine affected the levels of GABA or D-serine (Figure 7B,C).

**Figure 8:**
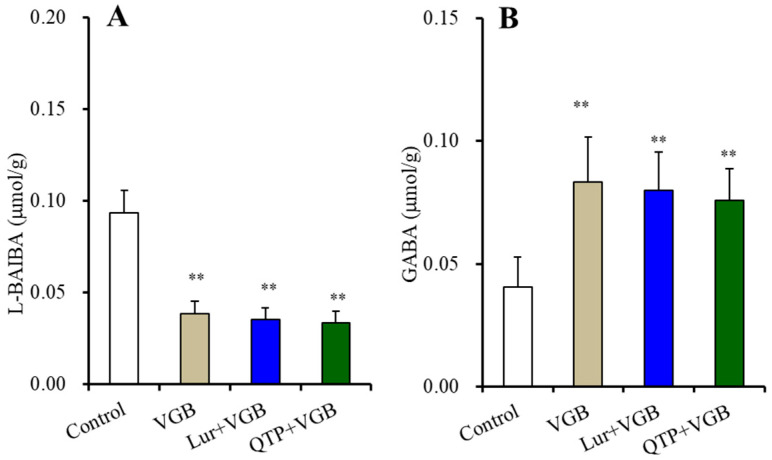
Interaction among chronic exposures of lurasidone (Lur: 500 nM for 14 d), quetiapine (QTP: 30 μM for 14 d), and vigabatrin (VGB: 200 μM) on intracellular levels of L-BAIBA (**A**) and GABA (**B**) in the astrocytes. Astrocytes were exposed to 200 μM vigabatrin alone (VGB), 200 μM vigabatrin with 500 nM lurasidone (Lur + VGB), 200 μM vigabatrin with 30 μM quetiapine (QTP + VGB) or vehicle alone (control: fDMEM containing 0.1% DMSO) for 14 d. Ordinate indicates mean ± SD (*n* = 6) of levels of L-BAIBA and GABA in the astrocytes (μmol/g protein). **: *p* < 0.01: relative to control by ANOVA with Tukey’s post hoc test.

Chronic exposure of 200 μM vigabatrin decreased and increased the levels of L-BAIBA and GABA, respectively (*p* < 0.01) (Figure 8A,B). After the chronic co-exposure of vigabatrin (200 μM) with lurasidone (500 nM) or quetiapine (30 μM), significant effects of 500 nM lurasidone and 30 μM quetiapine were not observed (Figure 8).

#### 3.3.2. Effects of Chronic Exposures of Lurasidone and Quetiapine on Second Messengers in the Astrocytes

**Figure 9:**
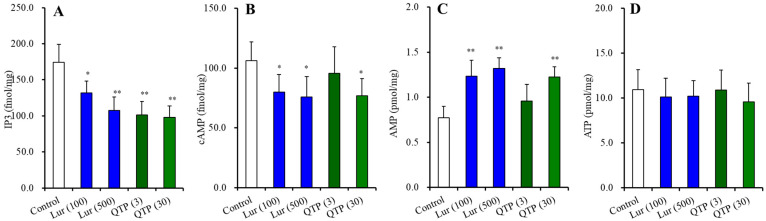
Effects of chronic exposures of lurasidone (Lur: 100 and 500 nM for 14 d) and quetiapine (QTP: 3 and 30 μM for 14 d) on intracellular levels of IP3 (**A**), cAMP (**B**), AMP (**C**) and ATP (**D**) in the astrocytes. Ordinate: mean ± SD (*n* = 6) of levels of IP3, cAMP (fmol/mg) and AMP, ATP (pmol/mg) in the rat cultured astrocytes. * *p* < 0.05, ** *p* < 0.01: relative to control by ANOVA with Tukey’s post hoc test.

Chronic exposures of lurasidone (100 and 500 nM for 14 d) (F(2,15) = 16.5 (*p* < 0.01)) and quetiapine (3 and 30 μM for 14 d) (F(2,15) = 27.7 (*p* < 0.01)) decreased the intracellular IP3 level in the astrocytes (Figure 9A). Chronic exposures of lurasidone also decreased the intracellular cAMP level (F(2,15) = 6.6 (*p* < 0.01)), whereas 30 μM quetiapine decreased but 3 μM quetiapine did not affect the cAMP level in the astrocytes (F(2,15) = 4.3 (*p* < 0.05)) (Figure 9B). Chronic exposures of lurasidone increased the intracellular AMP level (F(2,15) = 25.2 (*p* < 0.01)), whereas 30 μM quetiapine increased but 3 μM quetiapine did not affect the AMP level in the astrocytes (F(2,15) = 15.0 (*p* < 0.05)) (Figure 9C). Neither chronic exposures of lurasidone nor quetiapine affected the intracellular ATP level (Figure 9D).

#### 3.3.3. Astroglial L-BAIBA Release

The microdialysis study strongly indicated the possibility that L-BAIBA is a probable astroglial transmitter released through an activated hemichannel, whereas HKMRS-evoked stimulation (100 mM K^+^) is not a physiologically evoked stimulation [7,52,53]. To elucidate the mechanisms of astroglial L-BAIBA release, basal and ripple-evoked astroglial L-BAIBA releases were monitored [38].

**Figure 10:**
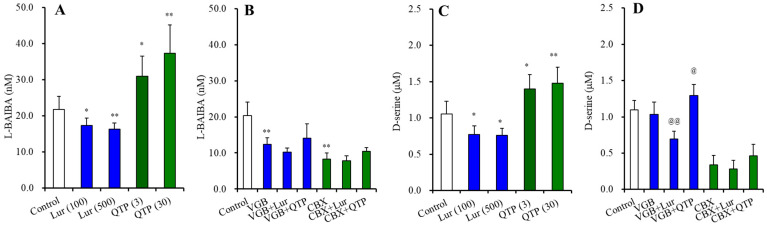
Concentration-dependent effects of chronic exposure lurasidone (Lur: 100 and 500 nM for 14 d) and quetiapine (QTP: 3 and 30 μM for 14 d) on astroglial ripple-evoked releases of L-BAIBA (**A**) and D-serine (**C**). Interaction among chronic exposures of antipsychotics (100 nM lurasidone and 3 μM quetiapine) and chronic exposure of 200 μM vigabatrin (VGB: ABAT inhibitor) or 100 μM carbenoxolone (CBX: astroglial hemichannel inhibitor) on ripple-evoked releases of L-BAIBA (**B**) and D-serine (**D**). In panels (**A**,**B**): Ordinate: mean ± SD (*n* = 6) of levels of L-BAIBA (nM). In panels (**C**,**D**): Ordinate: mean ± SD (*n* = 6) of levels of D-serine (μM). * *p* < 0.05, ** *p* < 0.01: relative to control by ANOVA with Tukey’s post hoc test. @ *p* < 0.05, @@ *p* < 0.01: relative to vigabatrin alone (VGB) by ANOVA with Tukey’s post hoc test.

During the resting stage, astroglial L-BAIBA release was not detected. However, ripple-evoked stimulation generated astroglial L-BAIBA release, and the ripple-evoked L-BAIBA release was inhibited by 100 μM carbenoxolone (Figure 10A,B). Chronic exposures of lurasidone (100 and 500 nM) (F(2,15) = 7.4 (*p* < 0.01)) and quetiapine (3 and 30 μM) (F(2,15) = 11.7 (*p* < 0.01)) decreased and increased ripple-evoked L-BAIBA release, respectively (Figure 10A). After chronic co-exposures of 200 μM vigabatrin with lurasidone or quetiapine for 14 d, the effects of lurasidone and quetiapine on ripple-evoked L-BAIBA release were not observed (Figure 10B). Under the inhibition of astroglial hemichannel activity by 100 μM carbenoxolone, the effects of lurasidone and quetiapine on ripple-evoked L-BAIBA release were also not observed (Figure 10B).

To clarify the mechanisms of these inhibitory effect of lurasidone and the stimulatory effect of quetiapine on ripple-evoked L-BAIBA release, effects of lurasidone and quetiapine on ripple-evoked releases of the established astroglial transmitter, D-serine, were determined. Similar to L-BAIBA, chronic exposures of lurasidone (100 and 500 nM) (F(2,15) = 7.5 (*p* < 0.01)) and quetiapine (3 and 30 μM) (F(2,15) = 7.8 (*p* < 0.01)) decreased and increased ripple-evoked D-serine release, respectively (Figure 10C). After the co-exposures of 200 μM vigabatrin with lurasidone or quetiapine, the ripple-evoked D-serine release remained decreasing and increasing, respectively (Figure 10D). However, under the inhibition of astroglial hemichannel activity by 100 μM carbenoxolone, the effects of lurasidone and quetiapine on ripple-evoked D-serine release were also not observed (Figure 10D).

#### 3.3.4. Interaction between Chronic Administrations of Quetiapine and SB269970 on AMPK Signaling

Our previous studies demonstrated that chronic exposure to quetiapine enhanced AMPK signaling; however, the activation of AMPK signalings by 3 μM quetiapine was larger than that by 30 μM [19]. These concentration-dependent biphasic effects of chronic administration of quetiapine on AMPK signalings are considered to be modulated by the combination between its high-affinity H1/5-HT2A receptors antagonism and low-affinity 5-HT7 inverse agonism. Therapeutic-relevant concentration of quetiapine (3 μM) activates AMPK signalings via H1/5-HT2A receptors blockade alone, whereas supratherapeutic concentration of quetiapine (30 μM) activates via the H1/5-HT2A receptor blockade but additively attenuates via 5-HT7 receptor inhibition, resulting in that AMPK signalings during exposure to 30 μM quetiapine are relatively suppressed compared to during exposure to 3 μM quetiapine [19]. To elucidate this hypothesis, interaction between chronic administration of quetiapine (3 and 30 μM) and 5-HT7 inverse agonist, 10 μM SB269970 on AMPK signalings in astrocytes were determined.

**Figure 11:**
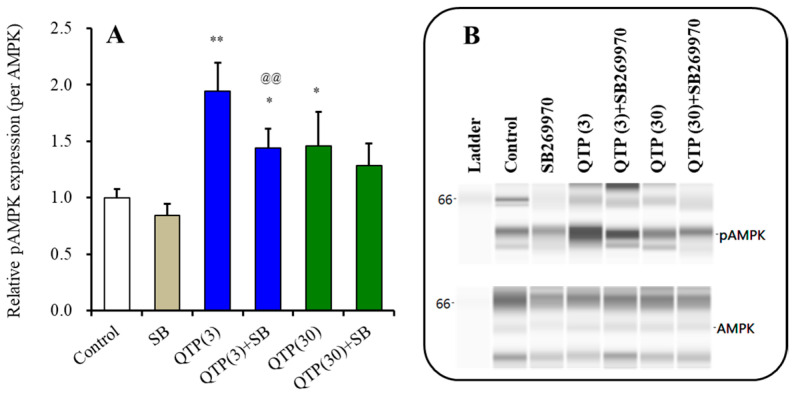
Interaction between chronic exposure (for 14 d) to therapeutic-relevant and supratherapeutic concentrations of quetiapine (QTP) and 10 μM SB269970 (SB) on phosphorylated AMPK (pAMPA) of cultured astrocytes. On the left-side histograms (**A**), ordinate: mean ± SD (*n* = 6) of the relative levels of pAMPK. * *p* < 0.05, ** *p* < 0.01: relative to control (drug free) and @@ < 0.01: relative to QTP (quetiapine: 3 μM) by one-way ANOVA with Tukey’s post hoc test. Right side panels (**B**) indicate their pseudo-gel images of capillary immunoblotting.

Chronic exposure to quetiapine (3 μM and 30 μM) enhanced astroglial AMPK signaling (Figure 11). Chronic exposure to 10 μM SB269970 alone did not affect AMPK signal-ling, but suppressed the stimulatory effects of therapeutic-relevant concentration of 3 μM quetiapine (Figure 11). However, SB269970 did not affect the AMPK signaling after the supratherapeutic concentration of quetiapine (30 μM) (Figure 11).

## 4. Discussion

### 4.1. Candidate Mechanisms of Weight Gain Associated with AMPK Signalings Induced by Antipsychotics

This study revealed that quetiapine increased L-BAIBA in the rat hypothalamus, similar to clozapine, whereas lurasidone conversely decreased the L-BAIBA level similar to brexpiprazole; however, neither quetiapine nor brexpiprazole affected D-BAIBA [11]. Initially, BAIBA enantiomers, D-BAIBA and L-BAIBA, were discovered in human urine in 1951 [54]. The detailed functions of BAIBA enantiomers had not been clarified for half a century, whereas recently, the function of the BAIBA enantiomer was identified as the protective myokine-like effects that regulate adipose tissue browning, enhanced insulin sensitivity, and suppressed obesity by a high-fat diet [16,17,47]. Notably, BAIBA enhances AMPK signaling under the endoplasmic reticulum stress [18]. It has been established that activation of AMPK signalings in peripheral organs plays important roles in the improving metabolic disturbances [3,12,47]; however, activation of hypothalamic AMPK signalings has been considered as major pathophysiological mechanisms of antipsychotics-induced weight gain and metabolic disturbances [3,6,11,12,47]. Indeed, high-risk antipsychotics for weight gain and metabolic complications, clozapine, olanzapine, and quetiapine suppress AMPK signalings in the liver [55], whereas these agents conversely enhance hypothalamic AMPK signalings [11,19,56]. Traditionally, the major mechanisms of antipsychotics-induced activation of hypothalamic AMPK signalings have been speculated to be the inhibition of H1/5-HT2A receptors [3,56]; however, the discrepant effects of clozapine and olanzapine on AMPK signalings between peripheral organs and central nervous systems could not be fully interpreted by the blockade of the histamine H1 receptor nor by the 5-HT2A receptor blockade alone [3,6,11].

The framework of established pathophysiology of atypical antipsychotics-induced weight gain and metabolic complication, 5-HT2A/H1 hypothesis, is complicated and composed of three processes. The first step is decreasing IP3 production via inhibition of the 5-HT2A receptor and H1 receptor. Decreased IP3 leads to secondary suppression of CICR via reduced IP3 receptor activation. Reduced CICR decreases ATP synthesis (or relatively increasing AMP/ATP ratio), resulting in activation of AMPK signalings [3,11,56]. Therefore, the present study confirmed whether atypical antipsychotics affect the complicated second messenger-mediated metabolic functional pathways associated with histamine H1 receptor or 5-HT2A receptor, according to the 5-HT2A/H1 hypothesis. Both quetiapine (high-affinity antagonist of the histamine H1 receptor and the 5-HT2A receptor) and lurasidone (high-affinity 5-HT2A receptor but low-binding affinity to the H1 receptor) decreased IP3 synthesis, whereas these two antipsychotics increased the hypothalamic intracellular AMP level without affecting the ATP level (relatively increasing AMT/ATP ratio). These results seem to support that both lurasidone and quetiapine affect the second messenger signaling pathway consistent with their receptor binding profiles; however, there are two contradictions as to the mechanism for weight gain and metabolic complication properties of both antipsychotics. Lurasidone, an antipsychotic with low-risk for weight gain and metabolic complication, actually suppresses hypothalamic AMPK activity [20], but, in this study, lurasidone elevated the AMP/ATP ratio, which activates AMPK activity. Dose–response curve analysis indicated that weight gain induced by quetiapine had a bell-shaped pattern and its peak dose was the highest dose of the therapeutic dose range [4]; however, in the present study, the dose-dependent effects of quetiapine on AMP/ATP ratio in the hypothalamus indicated that the therapeutic-relevant dose of quetiapine (10 mg/kg) did not affect the AMP/ATP ratio, but a supra-therapeutic dose of quetiapine increased the AMP/ATP ratio. These contradictions strongly indicate the existence of other mechanisms underlying the antipsychotics-induced weight gain and metabolic complication. So far, our previous studies have demonstrated that both clozapine and quetiapine, which are associated with high-risk for weight gain, enhanced AMPK signaling, whereas low-risk for weight gain antipsychotics, lurasidone and brexpiprazole, did not activate AMPK signalings [6,11,19,20,21]. These previous findings suggest that activation of AMPK signaling plays important roles in the weight gain and/or metabolic complication induced by antipsychotics [3,6,11]. Assuming that the 5-HT2A/H1 hypothesis suggests the existence of one of the metabolic pathways which activates AMPK signalings by antipsychotics, exploring the other metabolic pathway for activation of AMPK signalings induced by antipsychotics contributes to interpreting the actual pathophysiology of antipsychotics-induced weight gain and metabolic complication.

The function of BAIBA enantiomers has attracted attention as a therapeutic target for metabolic disturbance in peripheral organs, to compensate the functional abnormalities involved in the pathophysiology of metabolic disorders [16,17,18]. Especially, the BAIBA enantiomer is an established endogenous AMPK activator [47]. According to these previous findings, recently, we have already revealed that brexpiprazole and clozapine decreased the L-BAIBA level (in both extracellular and intracellular) in the hypothalamus, respectively, whereas these antipsychotics did not affect the plasma D-BAIBA level [11]. Therefore, to elucidate our hypothesis that L-BAIBA is a candidate molecule in the central nervous system contributing the pathophysiology of antipsychotics-induced weight gain and metabolic complication, the present study determined the effects of chronic administration of lurasidone and quetiapine on the L-BAIBA level and its associated signalings. In spite of the structural similarity between the BAIBA enantiomer, the metabolic pathways of L-BAIBA and D-BAIBA were independent each other. Indeed, D-BAIBA is synthesized from thymine and degraded by alanine-glyoxylate aminotransferase-2 [57], whereas L-BAIBA is synthesized from L-valine by ABAT [58,59,60]. The previous studies reported the inconsistent results regarding plasma D-BAIBA and L-BAIBA levels [61,62,63], whereas L-BAIBA is established to be the predominant BAIBA enantiomer in the central nervous system [11]. Indeed, the present study demonstrated that D-BAIBA and L-BAIBA are dominant enantiomers in the peripheral organs and central nervous system, respectively [11,63]. In the present study, neither lurasidone nor quetiapine did affect the amount of plasma BAIBA enantiomer level, due to D-BAIBA being the dominant enantiomer in the plasma. Contrary to a peripheral organ, in the hypothalamus, chronic administrations of lurasidone and quetiapine decreased and increased the amounts of the BAIBA enantiomer, respectively, via affecting the dominant BAIBA enantiomer, L-BAIBA. Therefore, these results indicate that quetiapine-induced weight gain and metabolic complication are probably involved in the enhancement of L-BAIBA signalings in the hypothalamus without affecting D-BAIBA signalings in the peripheral organs. Additionally, the mechanisms of low-risk for weight gain and metabolic complication of lurasidone can also be explained by its inhibitory effects on L-BAIBA in the hypothalamus.

Our previous and the present studies clearly demonstrated the biphasic dose-dependent effects of subchronic (for 7 d) and chronic (for 14 d) administration of quetiapine on AMPK signalings, since both 10 and 30 mg/kg/day of quetiapine increased AMPK signalings [19], whereas the increasing AMPK signaling by 30 mg/kg/day quetiapine was more modest than that of 10 mg/kg/day quetiapine. These results reasonably support the mechanisms of a bell-shaped increasing body weight by quetiapine; however, in the present study, both chronic administrations of 10 and 30 mg/kg/day for 14 d increased the body weight and L-BAIBA level in the hypothalamus, but the dose-dependent effects could not be observed. The discrepancies among the dose-dependent effects of chronic administrations of quetiapine on body weight, AMPK signaling, and L-BAIBA level suggest that increasing L-BAIBA induced by a therapeutic-relevant dose of quetiapine contributes to both increasing body weight and activation of AMPK signaling via increasing the L-BAIBA level in the hypothalamus, whereas mechanisms other than L-BAIBA possibly provide the attenuation of increasing body weight and AMPK signalings by a supratherapeutic dose of quetiapine compared to the therapeutic-relevant dose.

In this study, we hypothesised that 5-HT7 receptor inhibition was involved as the underlying mechanism of this discrepancy according to our previous demonstrations [6,11,19,20,21,27,64,65]. Lurasidone, a high-affinity 5-HT7 receptor inverse agonist (Ki = 0.5 nM), suppresses AMPK signalings [20,64]. Low dose/concentration of brexpiprazole did not inhibit AMPK signaling, but inhibition of supratherapeutic dose/concentration of brexpiprazole on AMPK signaling was observed [11,21]. These dose/concentration-dependent effects on AMPK signaling are spectated to be mediated by its 5-HT7 receptor binding affinity (Ki = 3.7 nM) [11,21,27]. The 5-HT7 receptor has been well known as the stimulatory receptor for cAMP synthesis via positively coupling to the Gs-protein [6,64]. Indeed, in spite of the high-affinity dopamine D2 receptor antagonist, in the present study, chronic administration of lurasidone decreased the cAMP level in the hypothalamus. These previous findings suggest that inhibition of the 5-HT7 receptor probably contributes to decreased AMPK signaling via inhibition of intracellular signaling associated with cAMP [6,11,20,21]. In the present study, the 5-HT7 inverse agonist, SB269970 attenuated the activation of AMPK signaling induced by chronic administration of therapeutic-relevant concentration of 3 μM quetiapine, whereas chronic administration of supratherapeutic concentration of 30 μM quetiapine enhanced AMPK signaling, but the activation was modest compared to therapeutic-relevant concentration of quetiapine. Furthermore, SB269970 could not inhibit the activated AMPK signalings induced by 30 μM quetiapine. Therefore, these results suggest that the therapeutic-relevant concentration of quetiapine activates AMPK signaling via inhibition of H1/5-HT2A receptors, whereas the stimulatory effects of supratherapeutic concentration of quetiapine is possibly suppressed by the low-affinity 5-HT7 inverse agonism of quetiapine. In other words, the dose-dependent bell-shaped pattern of quetiapine on weight gains was involved in the interaction of inhibition of 5-HT2A/H1 receptors and the 5-HT7 receptor. We speculated that the 5-HT7 receptor-mediated suppression of AMPK signalings is possibly involved in the relative suppression of the exchange protein directly activated by cAMP (EPAC) activity rather than PKA, since activations of PKA and EPAC suppress and enhance AMPK signalings, respectively [6,66]. The detailed mechanisms of regulation of AMPK signaling mediated by the 5-HT7 receptor should be determined in further studies.

### 4.2. Releasing Mechanisms as Candidate Gliotransmitter of L-BAIBA

It has been reported that BAIBA is a candidate endogenous agonist of glycine, GABA_A_ and GABA_B_ receptors [11,22,23]. Both in vivo microdialysis and cultured astrocytes studies demonstrated the detectable extracellular concentration of L-BAIBA, but could not detect those of D-BAIBA. The present study also demonstrated that L-BAIBA is a candidate gliotransmitter and is released to extracellular space via non-exocytosis mechanisms [11,22,23]. It has been established that exocytosis mechanisms are activated by 25–50 mM K^+^-evoked stimulation using microdialysis monitoring [50,67]; however, in the present study, conventional K^+^-evoked stimulation could not generate the depolarization-induced release of L-BAIBA. Therefore, the releasing mechanisms of L-BAIBA is not regulated by exocytosis. Contrary to conventional K^+^-evoked stimulation, the HKMRS-evoked persistent L-BAIBA release could be detected by microdialysis, suggesting that L-BAIBA is probably released via non-exocytosis processes [35,36,68,69]. Furthermore, HKMRS-evoked L-BAIBA release was drastically prevented by the hemichannel inhibitor, carbenoxolone, suggesting that L-BAIBA is released through a hemichannel.

Astroglial transmitter release is regulated by both exocytosis and non-exocytosis mechanisms [11,35,36]. We have already demonstrated that lurasidone and quetiapine suppressed and enhanced astroglial transmission, respectively, via modulating turnover of connexin43, which is a predominant subtype of the connexin family in astrocytes [6,19,20,28,30,64]. During the resting stage, the astroglial transmitter is not released through a hemichannel due to the low opening probability; however, electrical stimulation, such as depolarization, generates a persistent opening of the connexin43-containing hemichannel [38,69,70]. HKMRS (100 mM K^+^) evoked stimulation is a pathological stimulation, whereas ripple-evoked stimulation is a physiological stimulation. Clinical physiological studies identified the physiological importance of ripple-burst oscillations spreading among thalamus, hippocamps, and frontal cortex (80~250 Hz) on cognitive function, such as memory integration [71,72]. Indeed ripple-bursts are observed during the sleep-spindle burst using wide-band electrocorticogram [38,73,74]. The ripple-evoked stimulation conducted in the present study is reflected the physiological functions induced by electrical stimulation since the ripple-evoked stimulation was designed based on ripple-burst oscillations observed during the sleep-spindle bursts [38,75]. Indeed, ripple-evoked releases of both D-serine and L-BAIBA from cultured astrocyte were inhibited by carbenoxolone. Considering with previous preclinical findings, the present study, therefore, suggests that L-BAIBA is released through an activated astroglial hemichannel. In other words, L-BAIBA is a candidate astroglial transmitter. Additionally, L-BAIBA transmission probably contributes to cognitive function, such as memory integration associated with sleep since the ripple-burst increased astroglial L-BAIBA release.

The present study demonstrated that enhancement of intracellular signalings associated with L-BAIBA in the hypothalamus plays important roles in the antipsychotics-induced adverse reaction, such as weight gain and metabolic complication via activation of hypothalamic AMPK signalings; however, in other brain regions, L-BAIBA possibly contributes to cognition and memory integration. Therefore, the detailed significance of L-BAIBA transmission in the central nervous system should be elucidated by further studies.

## 5. Conclusions

The present study revealed that a GABA isomer, L-BAIBA, is a candidate gliotransmitter, and its potential functions in the central nervous system. L-BAIBA is a predominant BAIBA enantiomer in the central nervous system, and is selectively released through an activated astroglial hemichannel. Most clinically impactable functions of L-BAIBA in psychopharmacology are target molecules for weight gain and metabolic complication induced by several atypical antipsychotics, since L-BAIBA activates AMPK signaling in the hypothalamus, resulting in weight gain and metabolic disturbances. Indeed, a high-risk atypical antipsychotic for weight gain, quetiapine activated AMPK signaling in both hypothalamus and astrocytes via increasing L-BAIBA synthesis. Contrary to quetiapine, a lower-risk antipsychotic for weight gain, lurasidone suppressed L-BAIBA synthesis via possibly inhibition of 5-HT7 receptor. Contrary to mechanisms of adverse reaction, the present study demonstrated that L-BAIBA was released by ripple-burst, which plays fundamental roles in the cognition, such as memory integration. Therefore, the present study suggests that L-BAIBA is a candidate astroglial transmitter, and enhancement of L-BAIBA transmission probably exhibits a double-edged sword function on clinical actions and adverse reactions of several atypical antipsychotics.

## Data Availability

The data that support the findings of this study are available from the corresponding author upon reasonable request. Some data may not be made available because of ethical restrictions.
